# Elevated Ca^2+^ at the triad junction underlies dysregulation of Ca^2+^ signaling in dysferlin-null skeletal muscle

**DOI:** 10.3389/fphys.2022.1032447

**Published:** 2022-11-03

**Authors:** Valeriy Lukyanenko, Joaquin Muriel, Daniel Garman, Leonid Breydo, Robert J. Bloch

**Affiliations:** ^1^ Department of Physiology, University of Maryland School of Medicine, Baltimore, MD, United States; ^2^ Program in Biochemistry and Molecular Biology, University of Maryland, Baltimore, MD, United States; ^3^ Formulation Development, Regeneron Pharmaceuticals, Tarrytown, NY, United States

**Keywords:** CICR, Ca-induced Ca release, dysferlinopathy, GCaMP, BAPTA, injury, osmotic shock

## Abstract

Dysferlin-null A/J myofibers generate abnormal Ca^2+^ transients that are slightly reduced in amplitude compared to controls. These are further reduced in amplitude by hypoosmotic shock and often appear as Ca^2+^ waves (Lukyanenko et al., J. Physiol., 2017). Ca^2+^ waves are typically associated with Ca^2+^-induced Ca^2+^ release, or CICR, which can be myopathic. We tested the ability of a permeable Ca^2+^ chelator, BAPTA-AM, to inhibit CICR in injured dysferlin-null fibers and found that 10–50 nM BAPTA-AM suppressed all Ca^2+^ waves. The same concentrations of BAPTA-AM increased the amplitude of the Ca^2+^ transient in A/J fibers to wild type levels and protected transients against the loss of amplitude after hypoosmotic shock, as also seen in wild type fibers. Incubation with 10 nM BAPTA-AM led to intracellular BAPTA concentrations of ∼60 nM, as estimated with its fluorescent analog, Fluo-4AM. This should be sufficient to restore intracellular Ca^2+^ to levels seen in wild type muscle. Fluo-4AM was ∼10-fold less effective than BAPTA-AM, however, consistent with its lower affinity for Ca^2+^. EGTA, which has an affinity for Ca^2+^ similar to BAPTA, but with much slower kinetics of binding, was even less potent when introduced as the -AM derivative. By contrast, a dysferlin variant with GCaMP6f_u_ in place of its C2A domain accumulated at triad junctions, like wild type dysferlin, and suppressed all abnormal Ca^2+^ signaling. GCaMP6f_u_ introduced as a Venus chimera did not accumulate at junctions and failed to suppress abnormal Ca^2+^ signaling. Our results suggest that leak of Ca^2+^ into the triad junctional cleft underlies dysregulation of Ca^2+^ signaling in dysferlin-null myofibers, and that dysferlin’s C2A domain suppresses abnormal Ca^2+^ signaling and protects muscle against injury by binding Ca^2+^ in the cleft.

## Introduction

Dysferlin is an integral membrane protein of skeletal muscle that is missing in individuals with Limb Girdle Type 2B (LGMD2B), Miyoshi Myopathy (MMD1) and other, rarer muscular dystrophies (Aoki and Takahashi 1993; Urtizberea et al., 2008; Amato and Brown, 2011; Fanin and Angelini 2016). Studies of membrane repair in skeletal muscle have suggested that dysferlin plays a key role in the process (Bansal et al., 2003; Bansal and Campbell 2004; Ho et al., 2004; Glover and Brown 2007; Han and Campbell 2007), though its role may be secondary to that of other repair proteins (Lennon et al., 2003; Demonbreun et al., 2016; Demonbreun and McNally 2016; McDade et al., 2021). This is consistent with results from our laboratory showing that membrane repair after eccentric injury of skeletal muscle *in vivo* is not impaired by the absence of dysferlin (Roche et al., 2010), and that deficits in membrane integrity in dysferlin-null muscle are due in part to secondary effects associated with inflammation (Roche, et al., 2010; Roche et al., 2015). The subcellular localization of dysferlin, originally reported to be at the sarcolemma and in intracellular vesicles (Matsuda et al., 1999; Bansal et al., 2003; Cenacchi et al., 2005), has more recently been demonstrated to concentrate primarily in the transverse tubular membranes at triad junctions (Roche et al., 2011; Kerr et al., 2013), where the bulk of the protein is exposed to the sarcoplasm and the short C-terminal sequence is extracellular (Kerr et al., 2013).

This localization suggested to us that dysferlin might play a role in regulating Ca^2+^ signaling in healthy muscle, and that the dystrophic phenotypes that arise in its absence may be due in part to changes in the regulation of Ca^2+^, which are believed to be pathogenic in many diseases of skeletal muscle (e.g., Allen et al., 2010; Burr and Molkentin 2015; Kushnir et al., 2018; Mareedu et al., 2021). We tested this idea by studying the Ca^2+^ transients of dysferlin-null A/J myofibers in culture, before and after mild injury caused by a brief hypoosmotic shock. Our previous studies had shown that the transients generated in A/J fibers were ∼15% smaller than those in control myofibers or in fibers transfected to express dysferlin (Lukyanenko et al., 2017; Muriel et al., 2022). After osmotic shock, however, A/J fibers showed a precipitous drop in the amplitude of Ca^2+^ transients compared to controls. In addition, many shocked fibers showed spontaneous Ca^2+^ transients accompanied by Ca^2+^ sparks and waves, typically associated with Ca^2+^-induced Ca^2+^ release (CICR). These phenotypes were completely reversed by transfection of fibers to express dysferlin or by treatment of the fibers before and during hypoosmotic shock with drugs that block the L-type Ca^2+^ channels (LTCC; also known as dihydropyridine receptors, DHPR) and ryanodine receptors (RyR1) that mediate the release of Ca^2+^ (Lukyanenko et al., 2017). We interpreted these results to mean that dysferlin in healthy muscle suppresses CICR and that upon injury in the absence of dysferlin, CICR becomes an important contributor to Ca^2+^ signaling, potentially contributing to pathogenesis (Kerr et al., 2014).

Here we test this idea by examining the effects of a permeable form of BAPTA, a chelator with an affinity for Ca^2+^ of ∼160 nM. We incubated control and dysferlin-null A/J myofibers with very low concentrations of BAPTA-AM, insufficient to affect the levels of Ca^2+^ generated during a Ca^2+^ transient, and then studied the effects on the transients, before and after hypoosmotic shock injury. Our experiments show that, upon uptake into muscle fibers to a concentration we estimate at ∼60 nM, BAPTA-AM is sufficient to revert A/J fibers to the control Ca^2+^ signaling phenotype. We then tested two other Ca^2+^ chelators, Fluo-4 and EGTA, each introduced as their -AM derivatives. These reagents bind Ca^2+^ with lower affinity (Fluo-4) or slower kinetics (EGTA) and thus higher concentrations were needed to generate results similar to BAPTA’s. Finally, we examined myofibers expressing dysferlin containing GCaMP6f_u_ (Helassa et al., 2016) in place of its most N-terminal C2 domain (GCaMP6f_u_-DYSF-ΔC2A). This molecule targets the triad junction like wild type (WT) dysferlin and like WT dysferlin it supports Ca^2+^ signaling at WT levels. In comparison, GCaMP6f_u_ alone is less potent. Our results suggest that leak of Ca^2+^ into the triad junction underlies dysregulation of Ca^2+^ signaling in dysferlin-null myofibers. They further suggest that dysferlin’s ability to bind Ca^2+^ in the triad junction is sufficient to protect muscle from changes in Ca^2+^ signaling induced by injury or exercise.

## Materials and methods

### Ethical approval

All procedures involving mice complied with the *Guide for the Care and Use of Laboratory Animals* (NIH publication No. 85–23, revised 1996). Experimental protocols were approved by the Institutional Animal Care and Use Committee of the University of Maryland School of Medicine.

### Mice

Dysferlin-null (A/J) and control (A/JCr, C57Bl/6) mice were obtained from the Jackson Laboratory (A/J, C57Bl/6) or the National Cancer Institute, Frederick, MD (A/JCr) or bred at the University of Maryland, Baltimore (C57Bl/6). Mice were anesthetized with 2.5–4.5% isoflurane vaporized in oxygen and euthanized by cervical dislocation. Mice were 12–16 weeks of age at the time their tissues were studied.

### Plasmid constructs and transfection

mVenus-dysferlin (N-terminal Venus) (Addgene plasmid 29,768) (Covian-Nares et al., 2010) was provided by The Jain Foundation (www.jain-foundation.org). The constructs carrying Venus-dysferlin and Venus-dysferlin missing the C2A domain (residues 1–107) have been reported (Kerr et al., 2013; Muriel et al., 2022). For insertion of the GCaMP6f_u_ sequence (AddGene), we deleted the Venus moiety from Venus-dysferlin missing the C2A domain with NheI and KpnI and replaced it with GCaMP6f_u_ using the same restriction sites. This placed the GCaMP6f_u_ sequence in frame with the rest of the dysferlin ORF.

*In vivo* gene transfer *via* electroporation into FDB fibers was adapted from published methods (DiFranco et al., 2009), as described (Kerr et al., 2013; Lukyanenko et al., 2017; Muriel et al., 2022). Venus-dysferlin (V-Dysf), GCaMP6f_u_ and their variants were visualized in cultured myofibers (see below) with a Zeiss Duo Laser Scanning Confocal System (Carl Zeiss, Thornwood, NY), equipped with a C-Apochromat 40×/1.20 W Korr objective. Fluorescence excitation and emission were at 488 and >505 nm, respectively, with the laser intensity attenuated to 1%.

### Isolation of myofibers from FDB muscle

Mice were anesthetized and FDB muscles from both feet were harvested. A 2 week period was allowed after electroporation. Single myofibers were prepared in DMEM with 2% (wt/vol) BSA, 1 μl/ml gentamicin, and 2 mg/ml type II collagenase (Gibco, ThermoFisher, Waltham, MA) for 2 h at 37°C. Myofibers were kept for 12–14 h at 37°C and plated on 96-well plates coated with laminin (Sigma-Aldrich, St. Louis, MO) 1 h before experimentation. Fibers were then washed in normal Tyrode’s solution, pH 7.4, containing 140 mM NaCl, 5 mM KCl, 0.5 mM MgCl_2_, 0.3 mM NaH_2_PO_4_, 5 mM HEPES, 5.5 mM glucose, 1.8 mM CaCl_2._

### Confocal imaging

Isolated FDB fibers were loaded for 45 min at 37°C with 4.4 µM Rhod-2AM in the presence of 0.25% Pluronic F-127 (both from ThermoFisher), diluted in culture medium, then washed with Tyrode’s solution. When BAPTA-AM, Fluo-4AM or EGTA-AM was used, it was included in the same solution at concentrations from 0 to 250 nM.

Rhod-2 was excited with the 560 nm laser line with the intensity attenuated to 0.5% and emission was monitored at >575 nm with a LP 575 filter. Fluo-4 or GCaMP6f_u_ were excited by light at 488 nm (25 mW argon laser, intensity attenuated to 1%) and fluorescence was measured at wavelengths of >515 nm. Trains of voltage pulses transients were induced by field stimulation (1 Hz for 10 s) every 1 min for 5 min, as reported (Lukyanenko et al., 2017; Muriel et al., 2022). Perfusions and imaging were done in the dark.

Line-scan images were taken in the middle of myofibers at 1.9 ms per line at maximal aperture. ImageJ 1.31v (NIH, Bethesda, United States) averaged the profiles for every pixel over time and took the maximal value for each of 10 voltage pulses to calculate the mean maximal value of the Ca^2+^ transients, which were typically 225 pixels in width. The difference between maximal fluorescence intensity (*F*_max_) and background fluorescence (*F*_*o*_), normalized to *F*_*o*_. is reported.

As the amplitudes of the Ca^2+^ transients in electroporated myofibers are higher than those in controls (Lukyanenko et al., 2017), we analyzed results from electroporated and non-electroporated samples separately, with differences analyzed with the paired Student t-test for the former, to compare regions that expressed the transgene with regions that did not, and a simple *t* test for the latter.

### Osmotic shock injury

Osmotic shock injury (OSI) was induced as described (Lukyanenko et al., 2017). In brief, cultured FDB fibers were superfused with normal Tyrode’s solution and then for 60 s with a hypotonic Tyrode’s solution containing 70 mM NaCl. Cells were then perfused with isotonic Tyrode’s solution for 5 min. Experiments were performed at room temperature (21–23°C). Data were collected from myofibers cultured from 2 or more mice.

### Calibration of Fluo-4

Calibration was done in the glass-bottom chambers used for culturing myofibers. Solutions containing known concentrations of Fluo-4 were added in the solution (mM): 10 HEPES, 264.2 KCl, 5 EGTA, 2.7 CaCl_2_ (to yield [Ca^2+^]_free_ = 100 nM), 1.1 MgCl_2_ (to yield [Mg^2+^]_free_ = 1 mM), pH 7.4. The concentrations were calculated with WEBMAXC STANDARD. Line-scan images through the solution were taken at 1.9 ms per line at maximal aperture. Averaged data from 5 experiments were used to build the calibration curve. We used identical conditions to scan fibers preloaded with 10 nM Fluo-4AM.

### Immunoblotting

Immunoblotting for RyR used antibodies specific for RyR1 (ThermoFisher/Invitrogen), RyR2 (ProteinTech) and RyR3 (Millipore, ThermoFisher/Invitrogen), diluted 1:500. Gels were 3–8% Tris-Acetate with Tris-acetate-SDS running buffer (NuPAGE/Invitrogen). Secondary antibodies were HRP conjugates (anti-rabbit from Invitrogen; anti-mouse from Jackson ImmunoResearch), used at 1:10,000. Blots were visualized with SuperSignal West Femto Maximum Sensitivity Substrate (ThermoFisher) and imaged with a BioRad ChemiDoc MP instrument.

### Statistical analysis

Quantitative data are shown as mean ± SE. Statistical significance was determined with Student’s *t* test and *Χ* ^2^ analysis. A value of *p* < 0.05 was considered statistically significant.

### Materials

BAPTA-AM and EGTA-AM were from MilliporeSigma. Fluo-4AM was from Invitrogen. Unless specified otherwise, all other chemicals were from Sigma-Aldrich.

## Results

### BAPTA

**FIGURE 1:**
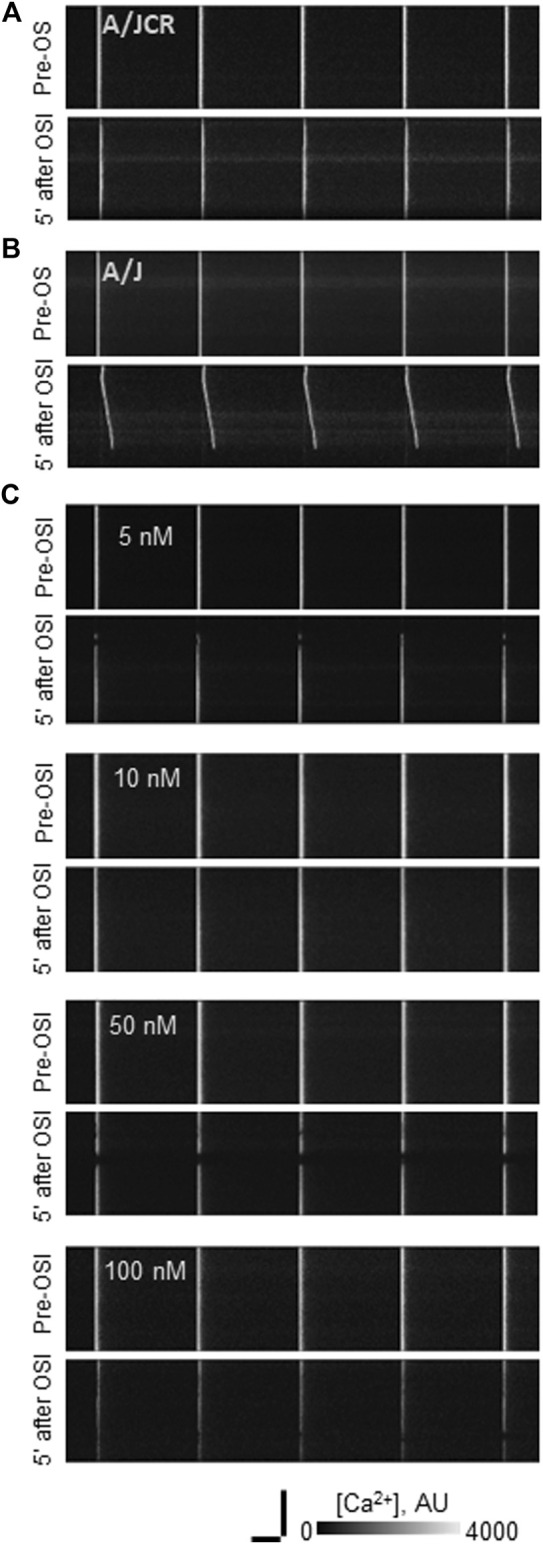
Effect of osmotic shock injury on Ca^2+^ transients in FDB fibers preloaded with different concentrations of BAPTA-AM. A/J or A/JCr myofibers were loaded with Rhod-2AM, with or without additional BAPTA-AM, subjected to 1 Hz stimulation, and imaged in line-scan mode under confocal optics (see Methods). Representative line-scan confocal images of voltage-induced Ca^2+^ transients in A/JCr **(A)** and sham (vehicle only) A/J [**(B)**: A/J] fibers and in A/J fibers preloaded with BAPTA-AM at 5, 10, 50 and 100 nM **(C)**. All examples in **(B,C)** were exposed to equal amounts of DMSO (1.5% by volume). All examples are shown before osmotic shock injury (OSI) and 5 min after OSI. Bars, 100 µm (vertical) and 250 ms (horizontal).

**FIGURE 2:**
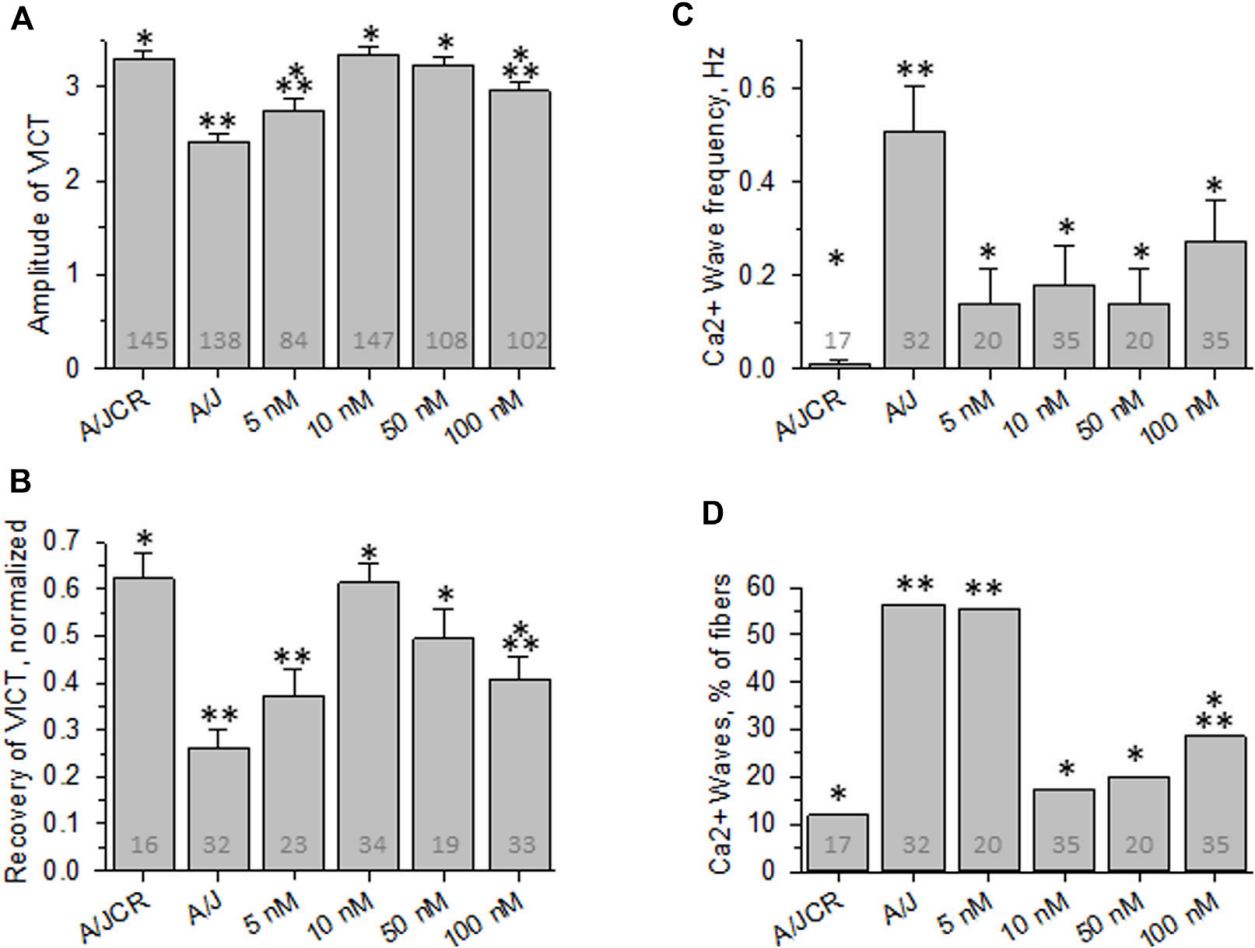
Effect of osmotic shock injury on Ca^2+^ transients in FDB fibers preloaded with different concentrations of BAPTA-AM. Data from experiments like those shown in Figure 1 were analyzed. **(A)**, averaged amplitudes of Ca^2+^ transients before OSI presented as (F_max_-F_0_)/F_0_. **(B)**, averaged data for recovery of Ca^2+^ transients at 5 min after OSI. **(C)**, averaged data for frequency of Ca^2+^ waves at 5 min after OSI. **(D)**, % fibers that produced Ca^2+^ waves at 5 min after OSI. N is indicated in each bar. *. *p* < 0.05 compared to A/J (sham). **, *p* < 0.05 compared to A/JCr control. Student’s *t* test was used for A-C; *Χ* ^2^ was used for **(D)**. VICT = Voltage-induced Ca^2+^ transient.

We first examined the effects of incubating dysferlin-null A/J myofibers with increasing concentrations of BAPTA-AM in the presence of 4.4 µM Rhod-2AM. We elicited Ca^2+^ transients with field stimulation and recorded the increase in Rhod-2 fluorescence, which tracks [Ca^2+^]_i_ (Lukyanenko et al., 2017; Muriel et al., 2022). Figures 1, 2 show that incubation with ≥100 nM BAPTA-AM reduces the apparent amplitude of the Ca^2+^ transient, perhaps because the BAPTA generated by cleavage of the -AM moieties in the sarcoplasm accumulates to levels high enough to compete with the Rhod-2 and reduce its fluorescence during the transient. By contrast, concentrations of BAPTA-AM of ≤50 nM increase the amplitude of the Ca^2+^ transient by ∼15% (Figures 1C, 2A), to levels seen in dysferlin-positive, control A/JCr myofibers. This difference is significant (*p* < 0.05). DMSO, the vehicle, had no effect in the absence of the chelator (2.40 ± 0.10, *n* = 138 vs. 2.57 ± 0.07, *n* = 280, A/J fibers with DMSO present vs*.* DMSO absent, respectively, *p* = 0.09). These results show that low concentrations of BAPTA-AM enhance the amplitude of Ca^2+^ transients in A/J muscle fibers, restoring them to control levels.

**FIGURE 3:**
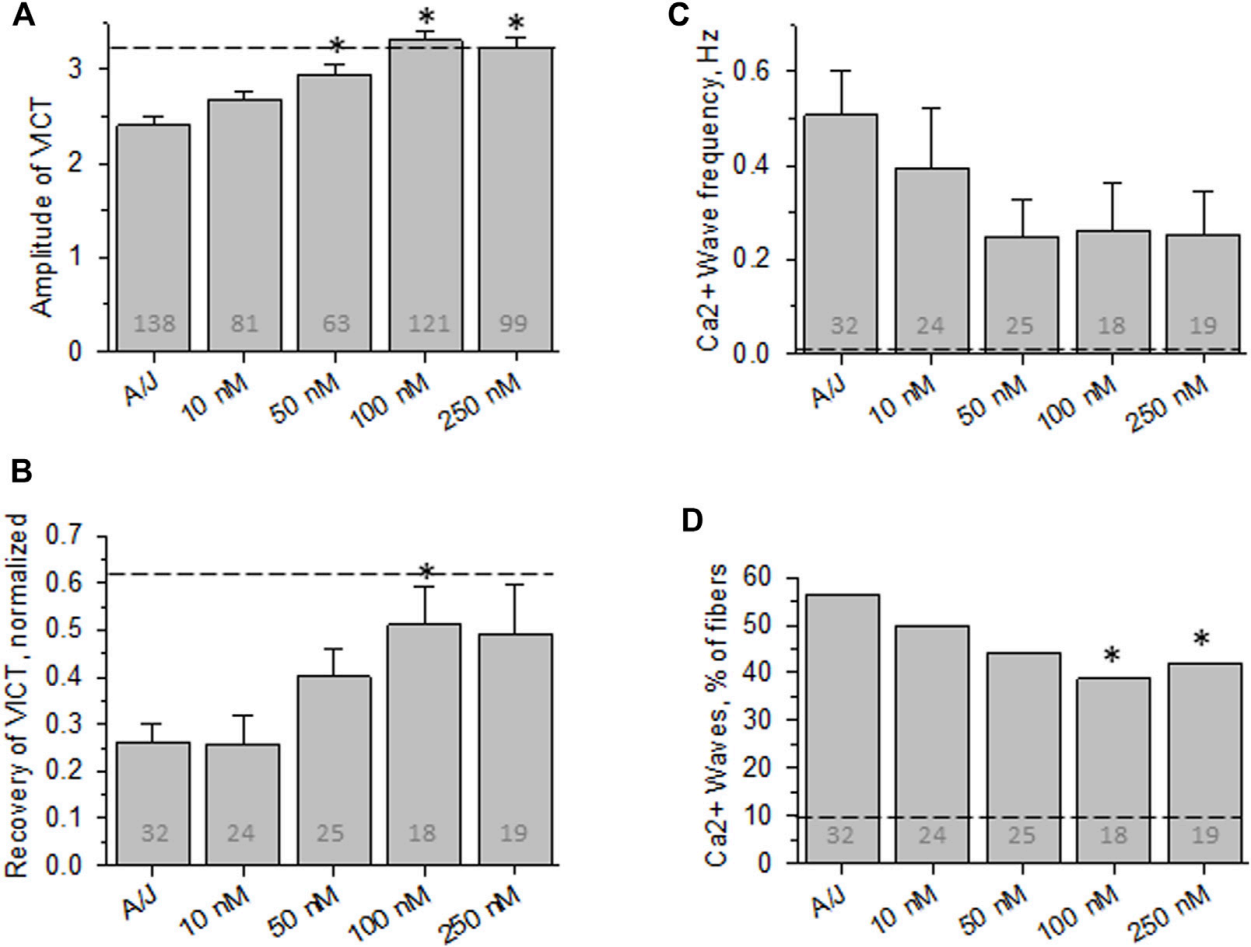
Effect of osmotic shock injury on Ca^2+^ transients in A/J FDB fibers preloaded with different concentrations of Fluo-4AM. As in Figure 2, but with myofibers loaded with Fluo-4AM. **(A)**, averaged amplitudes of Ca^2+^ release before OSI presented as (F_max_-F_0_)/F_0_. **(B)**, averaged data for recovery of Ca^2+^ transients at 5 min after OSI. **(C)**, averaged data for frequency of Ca^2+^ waves at 5 min after OSI. **(D)**, % fibers that produced Ca^2+^ waves at 5 min after OSI. Dashed lines represent values obtained with A/JCr fibers. *, *p* < 0.05 compared to A/J. N is indicated in each bar. Student’s *t* test was used for **(A–C)**; *Χ* ^2^ was used for **(D)**. VICT = Voltage-induced Ca^2+^ transient.

We next examined the effects of BAPTA-AM on the Ca^2+^ transients of dysferlin-null A/J myofibers after a brief osmotic shock injury (OSI). As above, we recorded the transients *via* Rhod-2 fluorescence in response to electrical stimulation. As we reported earlier (Kerr et al., 2013; Lukyanenko et al., 2017; Muriel et al., 2022), OSI significantly decreases the amplitude of Ca^2+^ transients of A/J fibers 5 min after injury, and the transients that appear are frequently accompanied by Ca^2+^ waves and spontaneous transients (Figures 1, 2B–D). In fibers incubated in 10 nM BAPTA-AM, however, the amplitudes of the transients were not reduced after osmotic shock injury (Figure 2B), and waves and spontaneous transients were reduced to control levels (Figures 1, 2C,D). These changes did not occur with DMSO alone (Figures 3B–D, A/J alone), suggesting that BAPTA mediates these effects.

We studied the concentration dependence of the inhibition of abnormal Ca^2+^ signaling as a function of BAPTA-AM concentration. We found that 5 nM BAPTA-AM was not sufficient to protect the transient against loss of amplitude after OSI, but that concentrations of 10 and 50 nM were effective (Figures 2A,B). These results suggest that the presence of the chelator was sufficient to maintain normal Ca^2+^ signaling following OSI, mimicking the activity of dysferlin in this assay.

We next measured the effects of the -AM derivative of Fluo-4, a variant of BAPTA that fluoresces upon binding of Ca^2+^. Although Fluo-4AM partitions into the sarcoplasm and is cleaved by intracellular esterases much like BAPTA-AM (Paredes et al., 2008; Smith et al., 2018), ten-fold higher concentrations were required to mimic the effect of BAPTA-AM on the recovery of the Ca^2+^ transient after OSI and to reduce the appearance of Ca^2+^ waves (Figures 3B–D). Even at 250 nM, Fluo-4AM failed to reduce Ca^2+^ waves to the levels seen with 10 nM BAPTA-AM (compare Figures 3D–2D). Thus, Fluo-4AM is less effective than BAPTA-AM, perhaps because its affinity for Ca^2+^ is ∼2-fold poorer (Paredes et al., 2008). Fluo-4 is also larger, carries a negative charge and has a dielectric constant that is 20-fold higher than BAPTA’s, and so may accumulate in the sarcoplasm less efficiently than BAPTA.

Despite the differences between Fluo-4AM and BAPTA-AM, we used the former to approximate the intracellular concentration of BAPTA needed to reduce the abnormalities in Ca^2+^ signaling that we routinely assay. We measured the fluorescence intensity of intracellular Fluo-4 in myofibers incubated with 10 nM Fluo-4AM under conditions identical to those we used with BAPTA-AM and compared it to a standard curve, with the assumption that the concentration of free intracellular Ca^2+^ is ∼100 nM (e.g., López et al., 1983; Harkins et al., 1993; Head 1993; Pressmar et al., 1994; Konishi and Watanabe 1995; Baylor and Hollingworth 2007). For the standard curve, we measured the fluorescence intensities at different concentrations of the K^+^ salt of Fluo-4 in the presence of 100 nM Ca^2+^ and under identical confocal imaging conditions (see Methods). The results indicate that Fluo-4 reached concentrations in the sarcoplasm of ∼60 nM, or about 6 times higher than its concentration in the bath (Supplemental Figure S1). Given the significant differences between the effects of BAPTA-AM and Fluo-4AM, BAPTA may accumulate to levels significantly higher than 60 nM, thereby buffering sarcoplasmic free [Ca^2+^] after OSI to ≤100 nM.

### EGTA-AM

**FIGURE 4:**
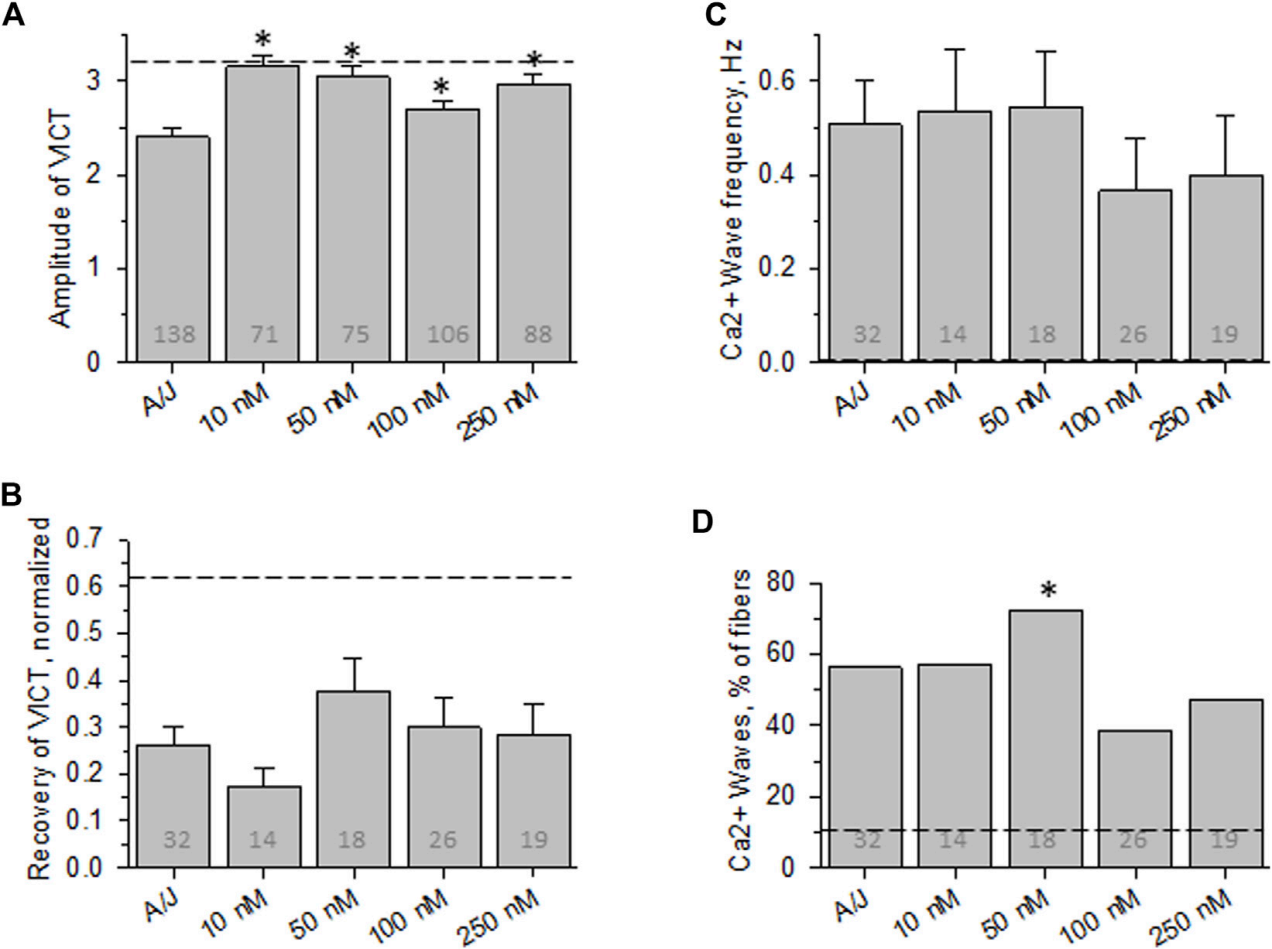
Effect of osmotic shock injury on Ca^2+^ transients in A/J FDB fibers preloaded with different concentrations of EGTA-AM. As in Figure 2, but with myofibers loaded with EGTA-AM. **(A)**, averaged amplitudes of Ca^2+^ release before OSI presented as (F_max_-F_0_)/F_0_. **(B)**, averaged data for recovery of Ca^2+^ transients at 5 min after OSI. **(C)**, averaged data for frequency of Ca^2+^ waves at 5 min after OSI. **(D)**, % fibers that produced Ca^2+^ waves at 5 min after OSI. Dashed lines represent values obtained with A/JCr fibers. *, *p* < 0.05 compared to A/J. N is indicated in each bar. Student’s *t* test was used for **(A–C)**; *Χ* ^2^ was used for **(D)**

Although the affinities of BAPTA and Fluo-4 for Ca^2+^ (in Mg^2+^-free conditions) are ∼160 and ∼370 nM, respectively, they both have relatively high “on” and “off” rates for Ca^2+^ and thus are able to chelate Ca^2+^ rapidly. The affinity of EGTA for Ca^2+^ is close to that of BAPTA, but its “on” and “off” rates for binding Ca^2+^ are ∼100-fold slower than BAPTA’s. We used EGTA-AM to test the idea that rapid, transient changes in the concentration of Ca^2+^, rather than changes in the equilibrium concentration alone, play a role in the abnormal Ca^2+^ signaling that we observe in A/J myofibers before and after OSI. Our results (Figure 4) show that incubating myofibers with concentrations of EGTA-AM up to 25-fold higher than the effective concentration of BAPTA-AM fails to protect the transient against disruption by OSI, although low concentrations are able to enhance the amplitude of the transient before injury. This suggests that the dysregulation of Ca^2+^ that alters the stability, but not the initial amplitude, of the Ca^2+^ transient in A/J fibers is due to rapid, transient changes in Ca^2+^ rather than the overall levels of Ca^2+^ in the cytoplasm.

### GCaMP6f_u_-DYSF-ΔC2A

Loading myofibers with BAPTA, Fluo-4 or EGTA, as we have done, can reduce the resting and peak levels of free Ca^2+^ in the sarcoplasm, which may only indirectly alter levels at the triad junction. We have postulated that abnormal Ca^2+^ signaling that follows injury of A/J myofibers is due to changes in Ca^2+^ at the triad junction that destabilize the LTCC-RyR1 couplons there, reducing normal Ca^2+^ release and promoting CICR. Here we test if suppression of local increases in Ca^2+^ at the triad junction is indeed sufficient to protect the Ca^2+^ transient from injury.

**FIGURE 5:**
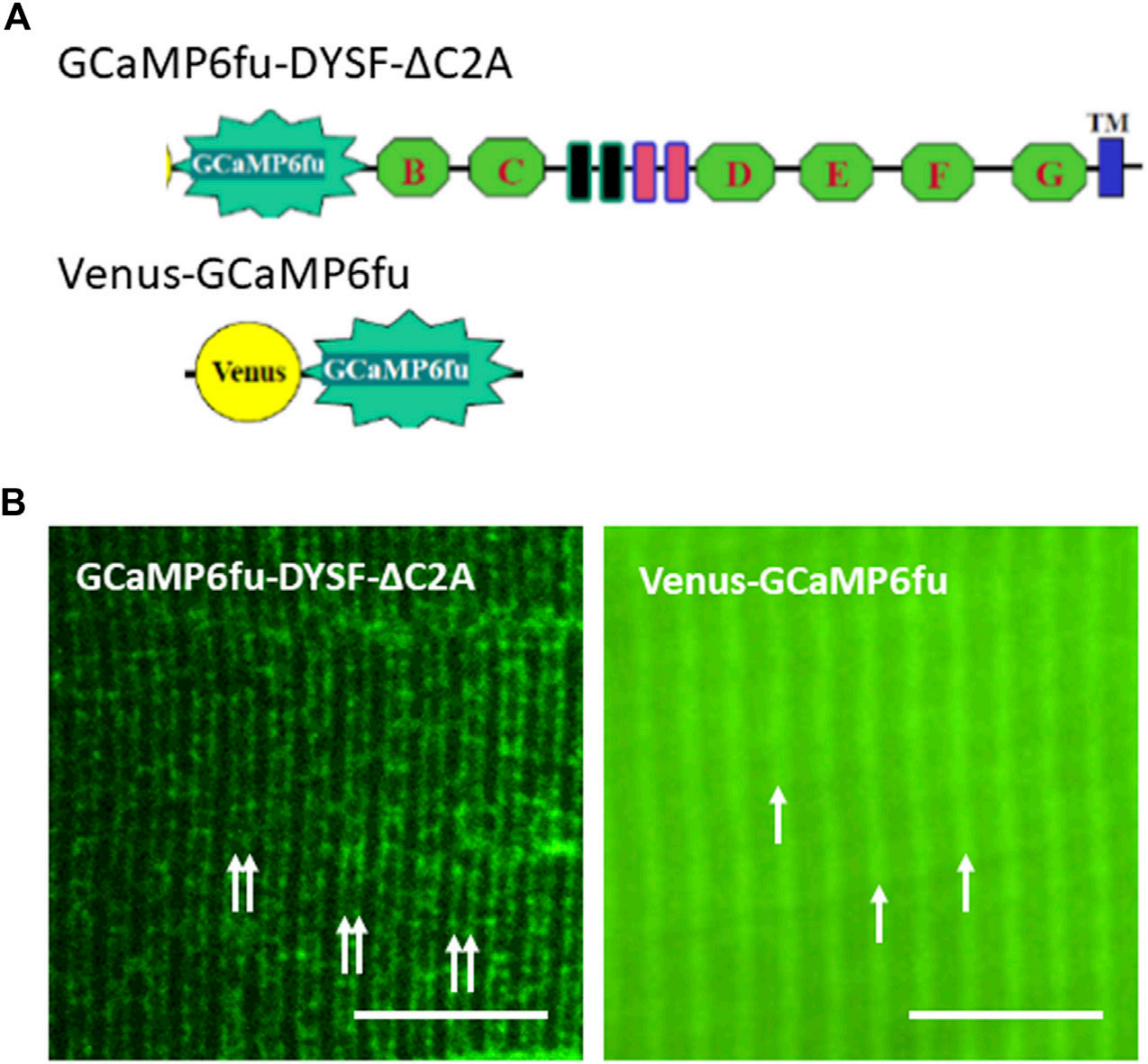
Distributions of GCaMP6f_u_ and GCaMP6f_u_-DYSF-ΔC2A in sarcoplasm. **(A)**. Cartoon diagrams of chimeric structures, which include the CMV promoter, the GCaMP6f_u_ reporter, and, for GCaMP6f_u_-DYSF-ΔC2A, the dysferlin ORF excluding most of the C2A domain (i.e., residues 108–2080) but including the remaining C2 domains B thought G (green hexagons), the Fer and DysF domains in the middle of the molecule (blue and pink outlined rectangles) and the transmembrane domain (blue rectangle near the C terminus; see Methods). **(B)**. Subcellular distribution of Venus-GCaMP6f_u_ and GCaMP6f_u_-DYSF-ΔC2A in transfected A/J myofibers. Plasmids were electroporated into A/J myofibers and imaged under confocal optics 2 weeks later. *Double arrows:* transverse tubules at level of triad junctions, as reported (Kerr et al., 2013; Muriel et al., 2022); *single arrows*: Z-disks, as reported (Muriel et al., 2022). Bars, 10 µm.

For these studies, we placed GCaMP6f_u_ at the N-terminus of Venus-dysferlin lacking the C2A domain (GCaMP6f_u_-DYSF-ΔC2A; Figure 5A). We chose this GCaMP variant because, like the C2A domain (Abdullah et al., 2014; Wang et al., 2021), GCaMP6f_u_ binds Ca^2+^ rapidly and with high affinity (Helassa et al., 2016). We found that, similar to the Venus construct of DYSF-ΔC2A, GCaMP6f_u_-DYSF-ΔC2A traffics normally to membranes at the level of the A-I junction (Figure 5B), concentrating in transverse tubules of the triad junction like both Dysf-ΔC2A and WT dysferlin (Muriel et al., 2022). By contrast, GCaMP6f_u_ expressed as a Venus fusion protein (Figure 5A) distributes much more uniformly in the sarcoplasm and, like GFP itself, only accumulates at the level of Z-disks (Figure 5B). When A/J fibers expressing GCaMP6f_u_ in the sarcoplasm or at the triad junction are loaded with Rhod-2 and electrically stimulated, both the GCaMP6f_u_ moiety and Rhod-2 register the changes in Ca^2+^ concentration. The signals generated by Rhod-2 were brighter than those generated by GCaMP6f_u_, but we were generally able to use either for measurements of transient amplitudes and waves.

**FIGURE 6:**
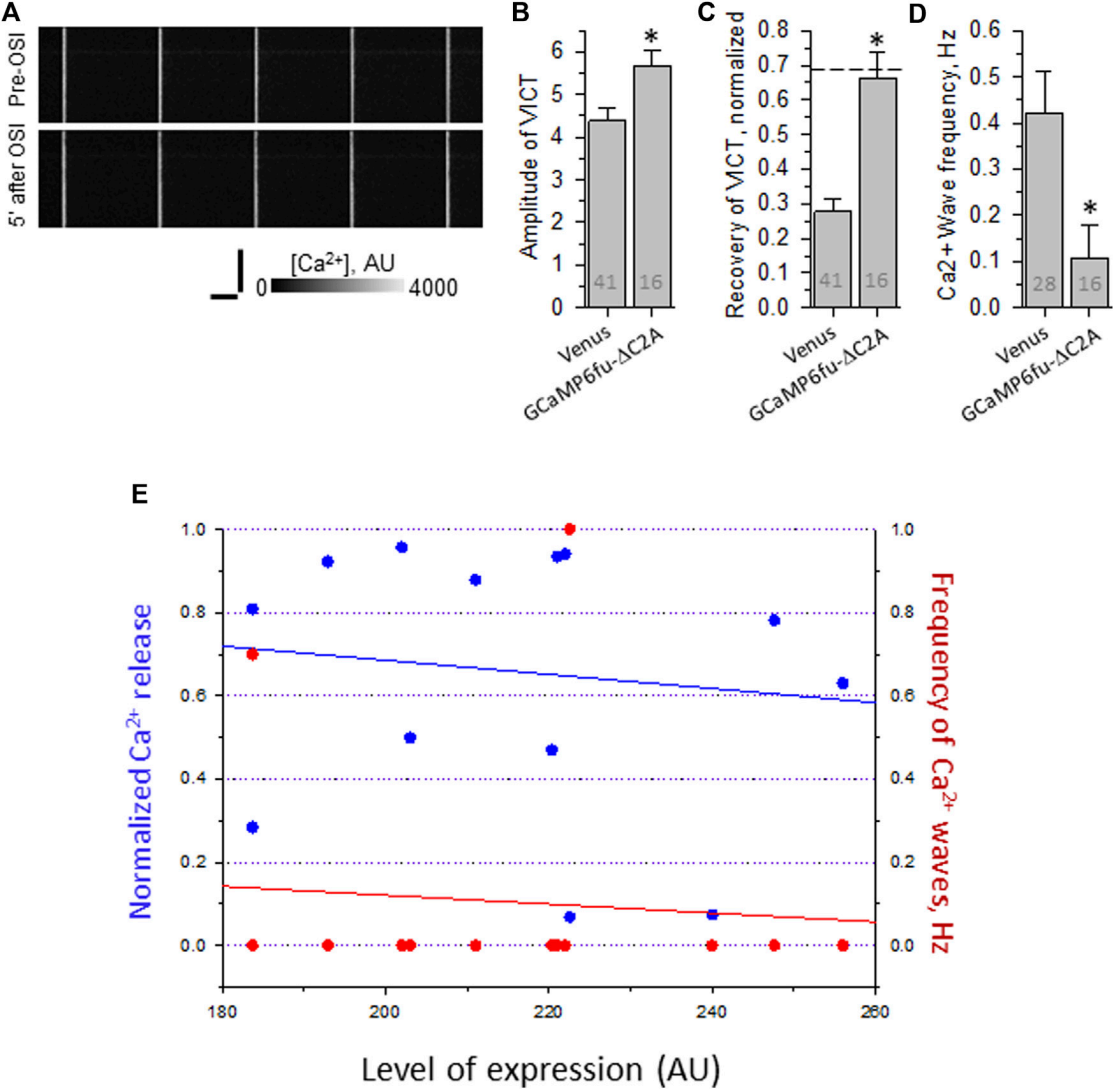
Effect of osmotic shock injury on Ca^2+^ transients in A/J FDB fibers transfected with GCaMP6f_u_-Dysf-ΔC2A. Myofibers were transfected by electroporation. Two weeks later, they were loaded with Rhod-2AM and assayed as in Figure 2. **(A)**, representative line-scan images of Ca^2+^ transients before and 5 min after OSI, as in Figure 1. **(B)**, averaged amplitudes of Ca^2+^ release before OSI for A/J fibers transfected with Venus or with GCaMP6fu-Dysf-ΔC2A. **(C)**, averaged data for recovery of Ca^2+^ transients from OSI for A/J fibers transfected with Venus or with GCaMP6fu-Dysf-ΔC2A at 5 min after OSI. Dashed line represents recovery in A/J fibers transfected with WT dysferlin. **(D)**, averaged data for frequency of Ca^2+^ waves at 5 min after OSI. N is indicated in each bar. Student’s *t* test was used for **(A–C)**; *Χ* ^2^ was used for **(D)** *, *p* < 0.05 compared to A/J fibers transfected with Venus. **(E)**. Recovery from OSI and frequency of Ca^2+^ waves as a function of GCaMP6f_u_-DYSF-ΔC2A expression. GCaMP6f_u_-DYSF-ΔC2A levels were determined in AU by measuring the intensity of the GCaMP6f_u_ fluorescence, after setting the background autofluorescence to 180 AU. For earlier data for A/J fibers expressing Venus- Dysf-ΔC2A, see Muriel et al., 2022.

Remarkably, fibers expressing GCaMP6f_u_-DYSF-ΔC2A show an increase in the amplitude of the Ca^2+^ transient similar to that observed with BAPTA and that seen when WT dysferlin is restored to dysferlin-null fibers (Figures 6A,B). (Please note that the amplitudes of the transients in uninjured myofibers subjected to electroporation are higher than those studied without electroporation, as reported Lukyanenko et al., 2017). Moreover, the transient after OSI remains at control levels, similar to fibers expressing WT dysferlin (Figures 6A,C). In addition, fibers expressing GCaMP6f_u_-DYSF-ΔC2A rarely show Ca^2+^ waves (Figure 6D). These results are consistent across a wide range in the level of expression of the fusion protein (Figure 6E), suggesting that it is acting in a limited volume, i.e., the triad junction.

**FIGURE 7:**
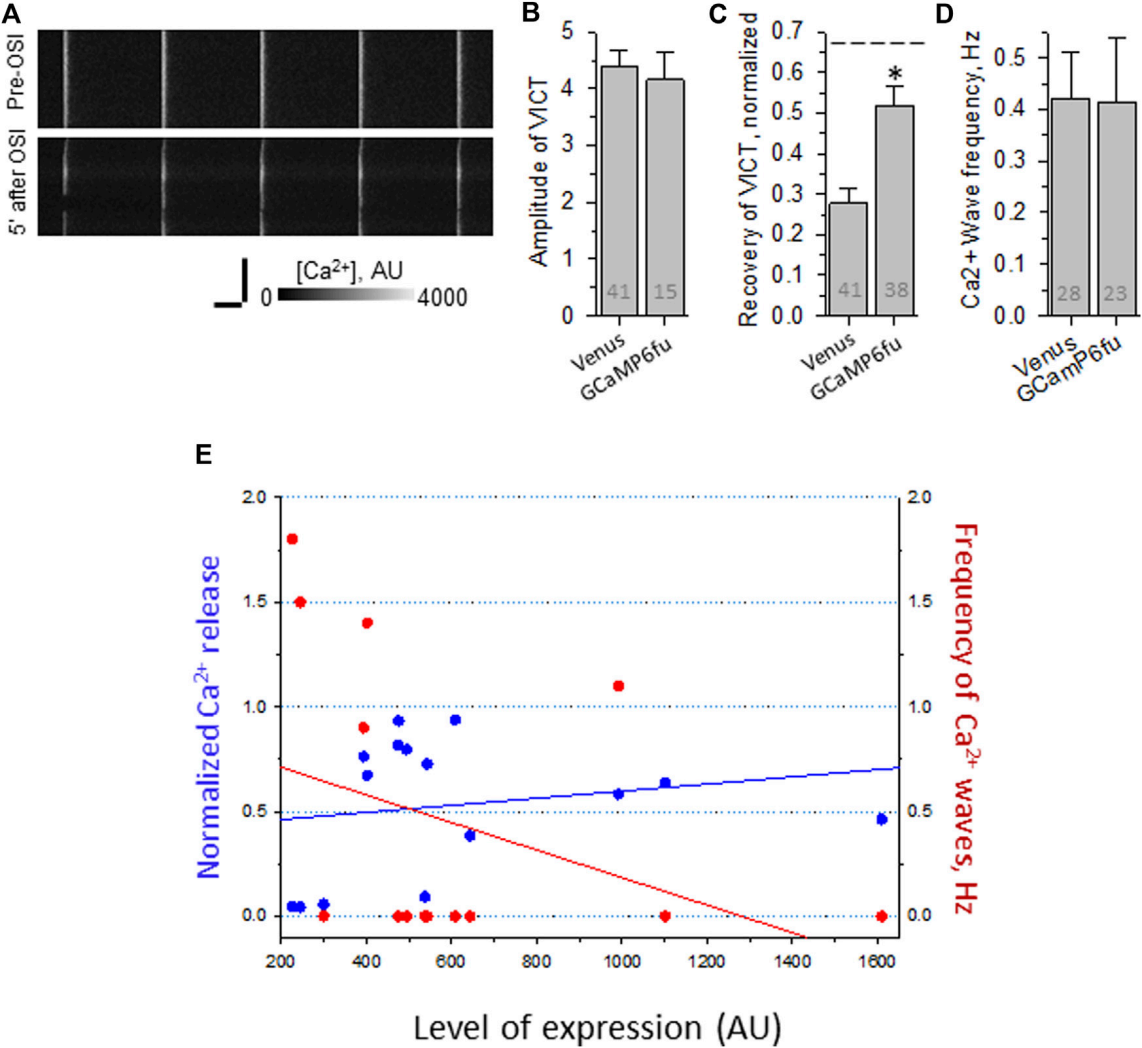
Effect of OSI on Ca^2+^ transients in A/J FDB fibers transfected with Venus-GCaMP6f_u_. Myofibers were transfected by electroporation. Two weeks later, they were loaded with Rhod-2AM and assayed as in Figure 2. **(A)**, representative line-scan images of Ca^2+^ transients before and 5 min after OSI. **(B)**, averaged amplitudes of Ca^2+^ release before OSI for A/J fibers transfected with Venus or with Venus-GCaMP6f_u_. **(C)**, averaged data for recovery of Ca^2+^ transients from OSI for A/J fibers transfected with Venus or with Venus-GCaMP6f_u_ at 5 min after OSI. The dashed line represents recovery in A/J fibers transfected with WT dysferlin. **(D)**, averaged data for frequency of Ca^2+^ waves at 5 min after OSI. N is indicated in each bar. Student’s *t* test was used for **(A–C)**; *Χ* ^2^ was used for **(D)** *, *p* < 0.05 compared to A/J fibers transfected with Venus. **(E)**. Recovery from OSI and frequency of Ca^2+^ waves as a function of Venus-GCaMP6f_u_ expression. Panels B–D show results obtained by imaging either Rhod-2 or GCaMP6f_u_ fluorescence. Panel E shows results obtained only with Rhod-2. Venus-GCaMP6f_u_ levels were determined in AU by measuring the intensity of the Venus fluorescence, after setting the background autofluorescence to 200 AU. For earlier data with A/J fibers expressing Venus alone, see Lukyanenko et al., 2017).

By contrast, GCaMP6f_u_ expressed as a Venus fusion protein fails to increase the amplitude of the transient in uninjured fibers (Figure 7B), and is somewhat less protective of the Ca^2+^ transients after OSI (e.g., Figures 7A,C). More strikingly, however, it fails to suppress the appearance of Ca^2+^ waves (Figure 7D). The differences with GCaMP6f_u_-DYSF-ΔC2A in amplitude and wave frequency after recovery from OSI were both statistically significant (*p* < 0.05). These results were far less consistent as a function of concentration than those of GCaMP6f_u_-DYSF-ΔC2A (Figure 7E; data obtained with Rhod-2, only). These results suggest that GCaMP6f_u_ in the sarcoplasm acts as a weak Ca^2+^ chelator and thus is considerably less effective than GCaMP6f_u_-DYSF-ΔC2A in the triad junction.

Our findings suggest that GCaMP6f_u_ localized by DYSF-ΔC2A to the triad junction, but not as a cytoplasmic protein, can serve the function of the C2A domain of dysferlin in Ca^2+^ signaling. Thus, one of dysferlin’s likely roles in maintaining the health of skeletal muscle is to bind Ca^2+^ at triad junctions and thereby protect the muscle from injury.

### Other ryanodine receptors

**FIGURE 8:**
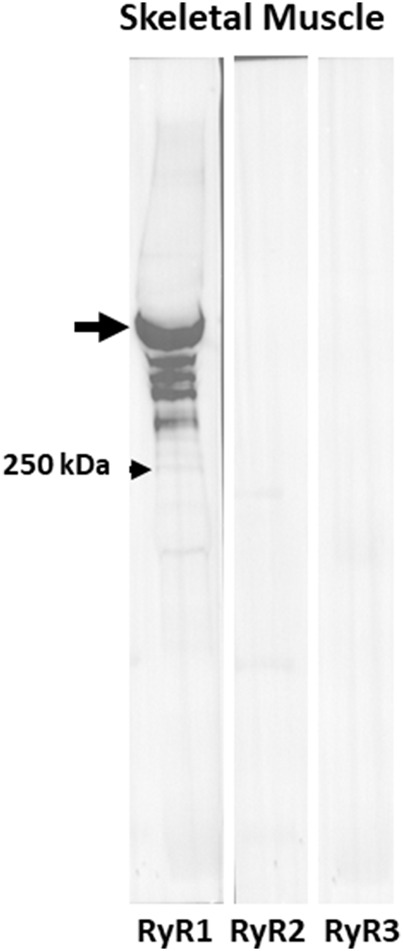
RyR1 but not RyR2 or RyR3 are expressed in fast twitch skeletal muscle. Immunoblots of extracts of *Tibialis anterior* muscles with antibodies to RyR1, RyR2, and RyR3. Only anti-RyR1 shows a strong band at ∼550 kDa.

As the presence of non-junctional RyR isoforms could account for CICR in A/J muscle fibers, we used immunoblotting of muscle extracts to determine if RyR2 and RyR3 were expressed together with RyR1. The blots of extracts of *Tibialis anterior* muscles from A/J mice, which like FDB fibers are primarily fast twitch, showed RyR1 to be present at high levels but RyR2 and RyR3 to be undetectable (Figure 8). They were readily detectable in heart and brain, however (not shown). This is consistent with several reports in GEO Profiles which show much lower levels of RyR2 and RyR3 mRNAs than RyR1 mRNA in *Tibialis anterior* muscles (see also Futatsugi et al., 1995; Marziali et al., 1996). Thus, RyR2 and RyR3 are unlikely to contribute significantly to abnormal Ca^2+^ signaling in dysferlinopathic muscle.

## Discussion

Previous reports from our laboratory showed that Ca^2+^ signaling is defective in dysferlin-null A/J muscle fibers, and that this phenotype can be reversed by the reintroduction of a Venus chimaera of WT dysferlin or by blocking the L-type Ca^2+^ channel (LTCC) and RyR1s (Lukyanenko et al., 2017). The defects include a small decrease in the amplitude of the Ca^2+^ transient in uninjured myofibers, a large decrease in transient amplitude in fibers injured by hypoosmotic shock, and the appearance of spontaneous Ca^2+^ transients, waves and sparks following shock. These features, which are typical of Ca^2+^-induced Ca^2+^ release (CICR), would be expected if the triad junctional regions of dysferlin-null muscle fibers experienced a small Ca^2+^ leak in the resting state that increased with injury. Although the leak in the resting state does not result in a detectable increase in [Ca^2+^]_i_ (Kerr et al., 2013), it has been measured and modeled in healthy in muscle (Cully et al., 2018; Barclay and Launikonis, 2022). An increase in resting leak could explain the lower amplitude of the Ca^2+^ transients we observe in A/J myofibers, due to a decrease in the amount of Ca^2+^ available for release upon electrical stimulation. Furthermore, additional increases in sarcoplasmic Ca^2+^ levels caused by hypoosmotic shock could evince spontaneous Ca^2+^ release events *via* CICR. We hypothesized that BAPTA, introduced into the sarcoplasm at low levels as the -AM derivative, would chelate the Ca^2+^ responsible for CICR and thereby reduce the effect of osmotic shock on the Ca^2+^ transient and suppress Ca^2+^ waves. Here we show that incubation of A/J myofibers with 10 nM BAPTA-AM is indeed sufficient to protect A/J myofibers against the loss of transient amplitude and to suppress waves. Consistent with this, the -AM derivatives of other Ca^2+^ chelators with reduced abilities to bind Ca^2+^ were less effective than BAPTA-AM. Our studies with the Dysf-ΔC2A-GCaMP6f_u_ chimera further suggest that the increase in Ca^2+^ that results in CICR-related dysregulation of Ca^2+^ signaling occurs primarily at or near the triad junction.

Our initial experiments utilized concentrations of BAPTA-AM that were considerably higher than 10 nM. These reduced the amplitude of the Ca^2+^ transients in uninjured fibers, measured with Rhod-2. It is likely that exposure of fibers to BAPTA-AM at concentrations ≥100 nM leads to sarcoplasmic concentrations of BAPTA that compete with Rhod-2 for free Ca^2+^ released following electrical stimulation. Remarkably, however, we found that concentrations of BAPTA-AM as low as 10 nM, much lower than concentrations used by other investigators (e.g., Jacquemond et al., 1991; Anderson and Meissner 1995; Gómez et al., 2006; Shkryl and Shirokova 2006; Ainbinder et al., 2015; Lamboley et al., 2015), were effective in suppressing abnormal Ca^2+^ signaling both before and after OSI.

As we could not directly measure BAPTA in the sarcoplasm, we used a close analog, Fluo-4AM, instead, and determined its concentration from its fluorescence after uptake, cleavage and equilibrium with sarcoplasmic Ca^2+^. We were obliged to perform these measurements with the aperture of our confocal microscope completely open, i.e., the identical conditions we used to observe myofibers, as we were unable to observe quantifiable fluorescence at low concentrations of Fluo-4 with the apertures consistent with confocal resolution. These conditions undoubtedly led to our inclusion of some out-of-focus fluorescence, too. Although we cannot compare the relative contributions to our measurements of out-of-focus fluorescence in myofibers and in solution, it is likely that our calibration, which indicates that Fluo-4 in the sarcoplasm reaches ∼60 nM in fibers incubated with 10 nM Fluo-4AM, underestimates the actual concentration of Fluo-4. This value should therefore be considered an estimate of the minimal concentration reached in myofibers exposed to 10 nM Fluo-4AM.

In contrast to Fluo-4AM, which carries a negative charge, BAPTA-AM is uncharged, suggesting that it should permeate the muscle cell membrane more efficiently. Nevertheless, if we assume that BAPTA accumulates like Fluo-4, and not considering the contribution of out-of-focus fluorescence to our calibration, intracellular concentrations of BAPTA should be ∼60 nM after myofibers are loaded with 10 nM BAPTA-AM. If the affinity of BAPTA for Ca^2+^ in the sarcoplasm is ∼160 nM (https://www.interchim.fr/ft/4/486103.pdf) and if we estimate [Ca^2+^]_I_ after hypoosmotic shock in the absence of BAPTA is ∼120 nM, then BAPTA would be expected to reduce the resting [Ca^2+^]_I_ after shock to ∼100 nM. A concentration of 120 nM is in the same range reported for the ability of Ca^2+^ to activate isolated RyR1, as well as RyR2 (Györke et al., 1994). CICR in the heart is also suppressed by BAPTA (Sham 1997) at concentrations sufficient to reduce [Ca^2+^]_I_ from 120 nM to <100 nM (Lukyanenko and Györke 1999). Our results are consistent with the idea that even low concentrations of BAPTA-AM can suppress CICR and Ca^2+^ waves in injured dysferlin-null myofibers.

Our results further indicate that BAPTA has a significant effect on the Ca^2+^ transient in uninjured A/J fibers. The reduced amplitude of the Ca^2+^ transient that we observe in A/J myofibers before injury or treatment with BAPTA is likely due to the activation of RyR1 by sarcoplasmic Ca^2+^ and the consequent small depletion of the luminal Ca^2+^ stores in the terminal cisternae of the sarcoplasmic reticulum, both of which would be inhibited by chelation of sarcoplasmic Ca^2+^ by BAPTA. Notably, murine muscle expresses undetectable levels of the two other major forms of the RyR, RyR2 and RyR3 (Figure 8), consistent with the idea that any effect on Ca^2+^ release is mediated by RyR1. As noted above, RyR1 has been linked to CICR-associated pathogenesis in other diseases of muscle (Endo 2009; see also Kushnir et al., 2018). Our results suggest that it is likely to contribute to pathogenesis in dysferlinopathies as well.

As CICR mediated by the RyR1 in skeletal muscle is thought to be suppressed when the channels are well ordered in LTCC-RyR1 couplons at triad junctions, the absence of dysferlin may well lead to a “couplonopathy” (Ríos et al., 2015; Ríos 2018), in which couplon organization is weakened, making it susceptible to further disruption upon hypoosmotic shock and thus enabling RyR1-mediated CICR in injured muscle. Others have reported that triad junctional architecture is altered in dysferlinopathy (Barefield et al., 2021).

Our evidence suggests that the couplon can be stabilized not only by uniformly chelating sarcoplasmic Ca^2+^ with BAPTA, but also by placing a mutant form of dysferlin with an enhanced ability to bind Ca^2+^ in the cleft of the triad junction itself. These experiments took advantage of our earlier observation that dysferlin lacking its N-terminal C2 domain, C2A, trafficked normally to the transverse tubules but did not support normal Ca^2+^ signaling (Muriel et al., 2022). Notably, the C2A domain has several binding sites for Ca^2+^ with affinities in the micromolar range {Abdullah et al., 2014, Wang et al., 2021). Although DYSF-ΔC2A prevents the generation of Ca^2+^ waves, it does not restore the amplitude of the transient to control levels and it fails to preserve the amplitude after OSI. We therefore used DYSF-ΔC2A as a backbone to target a Ca^2+^ binding protein moiety specifically to the junctional cleft by replacing C2A with GCaMP6f_u_, a calmodulin-based fluorescent Ca^2+^ indicator with rapid binding kinetics and high affinity for Ca^2+^ (0.89 μM at room temperature (Helassa et al., 2016). We find that, like BAPTA, GCaMP6f_u_ concentrated at triad junctions *via* Dysf-ΔC2A both enhances the amplitude of the Ca^2+^ transient to control levels in uninjured fibers and protects the transient from decreasing following OSI. GCaMP6f_u_ expressed as a soluble protein in the sarcoplasm is inactive in the former and trends to lower activity in the latter. It also fails to suppress Ca^2+^ waves. This strongly suggests that chelating Ca^2+^ within the triad junction of dysferlin-null skeletal muscle is sufficient to suppress abnormal Ca^2+^ signaling associated with CICR. Considered together with our other results, it further suggests that CICR is triggered primarily by the local elevation of Ca^2+^ in the junctional cleft and not by more widespread changes in [Ca^2+^]_i_.

The similarities in the effects of GCaMP6f_u_-DYSF-ΔC2A and WT dysferlin indicate that a key role of the C2A domain in native dysferlin in the triad junction may be to bind Ca^2+^ rapidly and with high affinity, consistent with earlier reports of the activity of the isolated C2A domain (Abdullah et al., 2014; Wang et al., 2021), and that this alone is sufficient to suppress abnormal Ca^2+^ signaling following injury in wild type muscle. This activity may help to explain the fact that healthy muscle but not dysferlinopathic muscle recovers quickly from exercise-induced injuries, avoiding necrotic fiber death (Roche et al., 2008; Roche et al., 2010; see also Roman et al., 2021).

Because DYSF-ΔC2A largely suppresses Ca^2+^ waves (Muriel et al., 2022), we do not know if placing GCaMP6f_u_ in the junctional cleft is sufficient to suppress waves. Further studies with additional constructs that also target the triad junction may be informative in this regard. Such studies may also permit us to use GCaMP-based methods to measure the concentrations of Ca^2+^ in the junctional cleft in healthy and dysferlin-null muscle, before and after injury.

## Data Availability

The original contributions presented in the study are included in the article/Supplementary Materials, further inquiries can be directed to the corresponding author.

## Supplementary material

Supplementary Figure S1 Calibration of [Fluo-4(K+)] in 100 nM free Ca^2+^. **(A)**, Difference in the background fluorescence in A/J fibers pretreated with 1.5% DMSO or 1.5% DMSO containing 10 nM Fluo-4AM. N is indicated in each bar. **(B)**, Calibration line for the 4 lowest concentrations of Fluo-4 (K+) of the 10 concentrations used for calibration. Open circle shows point for 23 AU. The same gain was used for each measurement. The solution contained (in mM): 5 EGTA, 2.7 CaCl2 (free, 100 nM), 1.1 MgCl_2_ (free, 1 mM), 10 HEPES, 264.2 KCl, pH = 7.4, super clean water. Calculations used WEBMAXC STANDARD.
