# Supplementary figures and images for: Elevated Ca2+ at the triad junction underlies dysregulation of Ca2+ signaling in dysferlin-null skeletal muscle
Source: Front Physiol. 2022 Nov 3;13:1032447. doi: 10.3389/fphys.2022.1032447 (PMC9669649; doi:10.3389/fphys.2022.1032447)

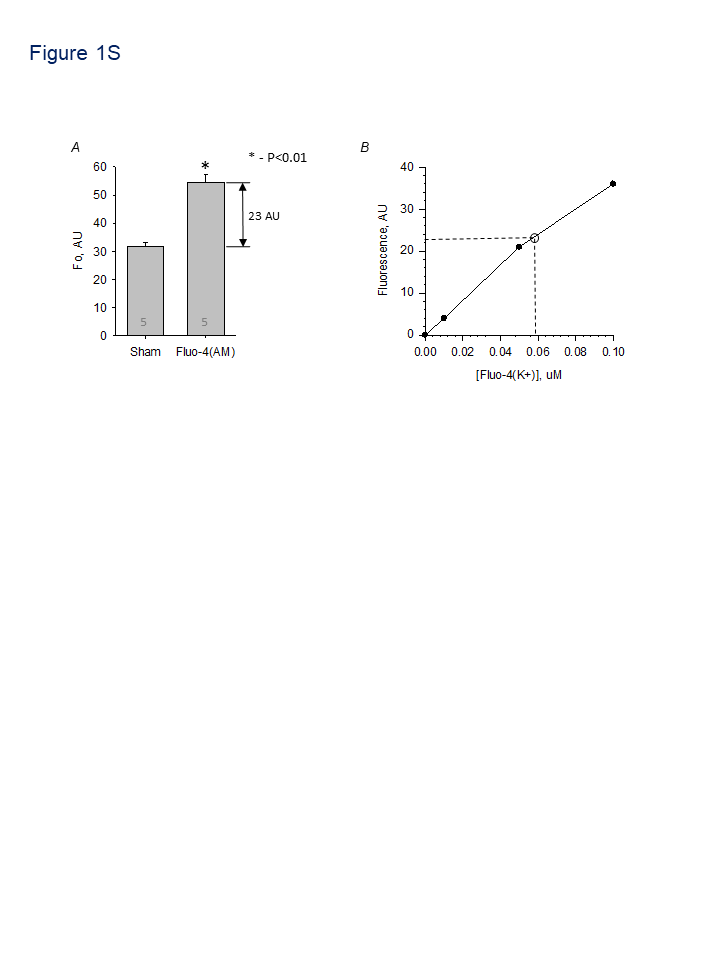

Supplement: Supplementary file 1 [file Image1.TIF]
